# *Entamoeba histolytica*: Five facts about modeling a complex human disease in rodents

**DOI:** 10.1371/journal.ppat.1008950

**Published:** 2020-11-12

**Authors:** Carolina Mendoza Cavazos, Laura J. Knoll

**Affiliations:** University of Wisconsin-Madison, Madison, Wisconsin, United States of America; University at Buffalo School of Medicine and Biomedical Sciences, UNITED STATES

## Fact 1: Rodent models do not mimic the entire life cycle of *E*. *histolytica*

*Entamoeba histolytica* is an extracellular enteric eukaryotic parasite. Globally, an average of 50 million cases and 55,000 to 100,000 deaths are due to *E*. *histolytica* infection each year, primarily impacting the developing world [1,2]. The world is widely unprepared for an outbreak of *E*. *histolytica* due to the lack of a vaccine and the use of a single drug type as treatment (reviewed in [3]). *E. histolytica* is the causative agent of the diarrheal disease known as amebiasis, but it can sometimes penetrate the intestinal wall, enter the circulation, and cause abscesses throughout the body, most commonly in the liver. *E. histolytica* has 2 main stages during its life cycle: the trophozoite and the cyst stage. The infectious agent is the cyst and is transmitted through the oral–fecal route via contaminated food or water. Animal models are not available for this stage interconversion, which is essential for disease propagation and pathogenesis. Excystation is the transition from the cyst form to the rapidly dividing trophozoite stage. Once a trophozoite, *E*. *histolytica* can either undergo invasive (5% to 10% of symptomatic infections) or noninvasive disease progression. As the parasite replicates during a noninvasive infection, it undergoes a second developmental change known as encystation, which involves the synthesis of a cyst wall to endure the exterior environment until a suitable host is encountered.

Because the cyst stage of *E*. *histolytica* cannot be induced in culture or rodents, *Entamoeba invadens* has served as an excellent model for the study of excystation and encystation in vitro. Some examples of insights from the study of *E*. *invadens* include the identification of stage-specific promoters [4], cholesteryl sulfate impact on encystation efficiency [5], the negative regulation of encystation by heat shock protein 90 (Hsp90) in vitro [6], and the discovery of various transcription factor that regulate stage conversion [7,8]. A rodent model that produces *E*. *histolytica* cysts could be used to generate new treatments or vaccines for amebiasis by targeting parasite development. However, tremendous advances have been accomplished using the current infection model including vaccine development (reviewed in [9]), microbiota–parasite interactions (reviewed in [10]), host innate and adaptive immune response to infection (reviewed in [11]), and molecular mechanisms responsible for tissue damage (reviewed in [12]).

## Fact 2: Trophozoites can initiate infection in rodent models

A robust way to culture trophozoites has been developed in the laboratory setting [13] and has been the primary resource to perform in vivo infection. Trophozoites are surgically delivered into the target organ within the animal models. For intestinal studies, the trophozoites are injected into a surgically exposed cecum or an artificial colonic loop. While this procedure is invasive and bypasses the oral portion of the infection, the murine model has provided insight into many parasite–host interactions. These discoveries include, but are not limited to, alteration to Paneth cell function by *E*. *histolytica* infection [14], the genetic predisposition to disease progression due to host polymorphism in leptin receptors [15] or intergenic insertion previously linked to inflammatory bowel disease [16], the ability to nibble on alive host cells (known as trogocytosis) [17], the discovery of pathogenicity of *Entamoeba moshkovskii* [18], and microbiome-mediated immune cell recruitment during *E*. *histolytica* infection [19,20]. To model invasive disease such as amoebic liver abscesses, trophozoites are delivered via intraportal inoculation to either hamsters or guinea pigs (reviewed in [21]). Physiochemical factors encountered throughout the gastrointestinal tract can influence parasite development but are bypassed using these methodologies [22]. To our knowledge, there are 3 studies conducted in the 1980s in which oral inoculation was performed with either cysts [23] or trophozoites [24,25]. *E*. *histolytica* strain SAW 408 trophozoites were orally inoculated into 3 types of rats pretreated with an antihistamine called cimetidine that blocks the production of stomach acid [24]. The rats displayed the expected pathology in the intestinal tract 21 days postinfection. However, mice that were previously described as SAW 760– (*Entamoeba dispar*) or SAW 408–sensitive [26] did not display pathology by histology or shedding by wet mounts. A follow-up study [27] tracked the health of rats 12 months after oral infection, concluding that infections can persist long term. These studies highlight that rodents can be useful for studying the complex biology of *E*. *histolytica* but that more research is needed.

## Fact 3: *E*. *histolytica* invasive disease can be modeled in rodents

*E*. *histolytica* disease outcomes range widely in humans: death, sepsis, liver abscesses, ulcers, dysentery, abdominal pain and mucoid diarrhea with occasional blood, and asymptomatic shedding. The field’s focus on invasive disease is due to the pathogenicity of the trophozoite stage within the host and the morbidity and mortality that invasive disease causes. One of the most widely used strains, HM-1:IMSS, was isolated from a male patient admitted into a public hospital in Mexico City in 1967. Although the virulence of the initial isolate was high, this strain undergoes continuous culture passaging, so virulence has changed over the years. Some laboratories pass trophozoites through the cecum or liver of animal models to retain virulence [28,29]. Maintaining virulence in this strain has allowed modeling of invasive disease. Frontline researchers in the amebiasis community have contributed immeasurable knowledge using intestinal pathology and development of liver abscess as a measure of disease progression. Hamsters and guinea pigs are used for the study of invasive disease, especially for the development of liver abscesses, and are the preferred model for vaccine development [9]. Various murine strains have been studied to determine what dictates susceptibility to *E*. *histolytica* infection, including but not limited to mice with a deletion in the secretory mucin of the gastrointestinal tract (MUC2) to determine the effect of an induced dysbiosis state on infection outcome [30] and mice with a deletion in the leptin receptor (Lep^ob^) to determine the molecular mechanism by which leptin modulates mucosal protection [31]. However, whether cysts are present in the feces is an outstanding question that has not been addressed.

## Fact 4: Disease modeling in rodents has shown microbiome–host–parasite interactions

Recent literature suggests that the host microbiome is a modifier of disease outcome and parasite development. The microbiota serves as an immune response trainer [32], processor of carbon sources unavailable to the parasite [33], sustenance to the parasite via phagocytosis [34], can produce metabolites that inhibit encystation [35], and together with a healthy mucus barrier, is the first line of defense against parasite infection (reviewed in [36]). Moreover, the microbiota is required for *E*. *histolytica* pathogenicity, as germ-free mice have an attenuated response to parasite infection [30]. The importance of the microbiome is also observed in human cohorts and case studies, demonstrating a correlation between dysbiotic state, a well-known niche for opportunistic pathogens, and *E*. *histolytica* infection outcome [37,38]. Recently, the microbiome has been shown to have an effect on disease severity via the recruitment of neutrophils [19,20]. Additionally, halting of encystation has been observed in *E*. *invadens* via microbiome metabolites [35]. The oral infection route is a modulator of disease progression and host immunological response, based on the impact pathogen delivery has in other parasites. For *Toxoplasma gondii*, immune murine knockout strains only displayed an increased susceptibility to infection when parasites were delivered through the natural route of infection [39]. For *Trypanosoma cruzi*, oral versus gastrointestinal delivery of trypomastigotes displays distinct patterns of disease progression in BALB/c mice [40]. Lastly, vaccine administration factors are evidence of how the route of delivery of antigens can lead to distinct immune responses, for example, mucosal inoculation leading to immunoglobulin A (IgA) production [41]. While bacterial microbiome changes have been correlated with various parasitic infections in numerous cohort studies (reviewed in [42]), it is unclear if the parasitic infection causes a state of dysbiosis or vice versa. Animals colonized with fixed microbial communities and orally challenged with either parasite stage will provide insight regarding the effect of the microbiome on parasite development.

## Fact 5: Developing a rodent model that produced cysts would be beneficial for the parasitology field

**Fig 1:**
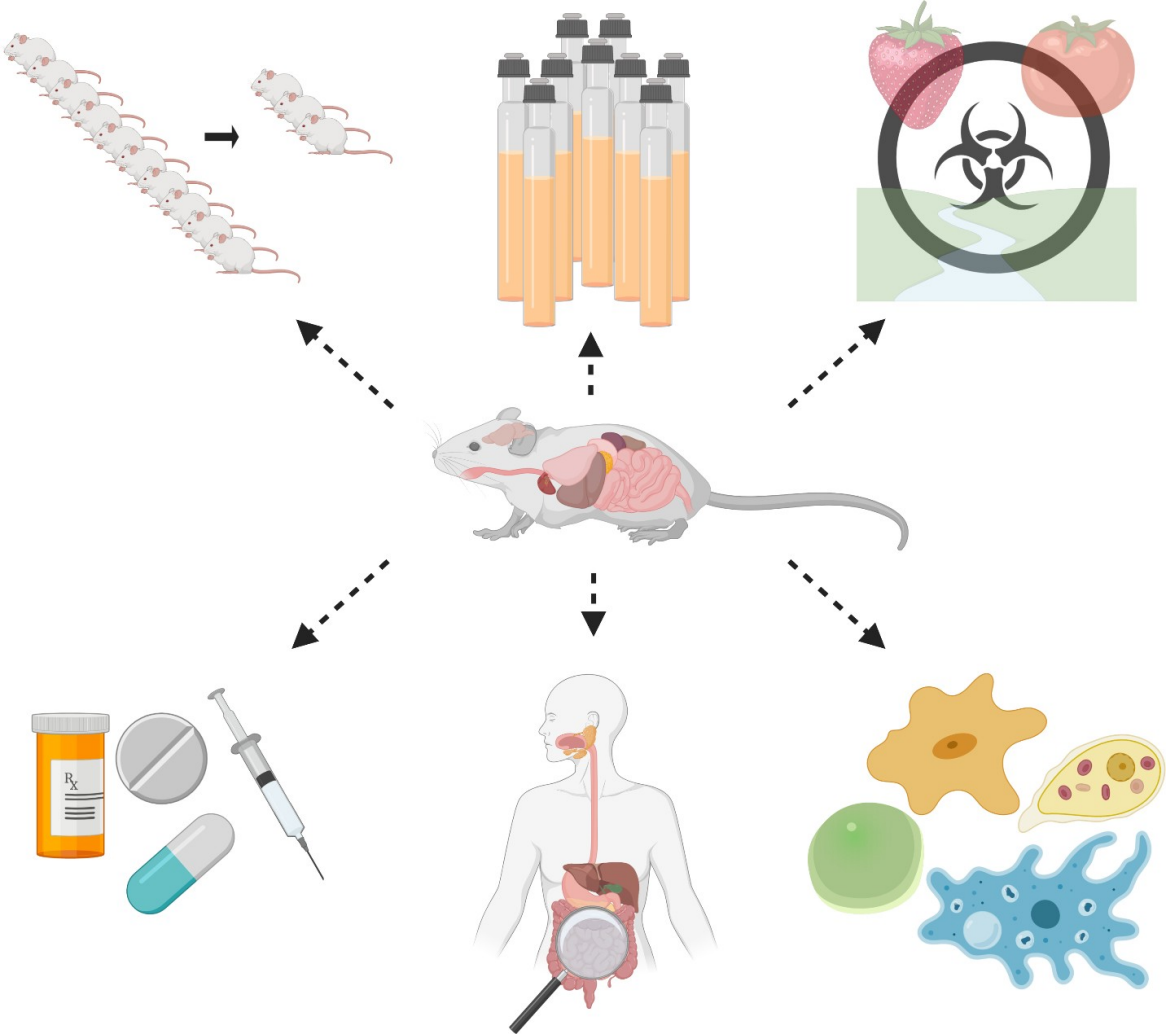
Ideal animal model that can support the full life cycle of *Entamoeba histolytica*. From top left to bottom right: (1) fewer animals used to maintain virulence; (2) less need for continuous passaging; (3) new detection methods for food safety and patient diagnostics; (4) model for drug screening; (5) focus on host response to developmental changes of the parasite in vivo; and (6) could serve as potential model to pioneer in vivo studies of *E*. *histolytica* with other pathogenic and nonpathogenic protozoans. Figure created with BioRender.com.

Trophozoites are the metabolically active form of *E*. *histolytica*, but the transmissible form is the cyst. These 2 forms of the parasites are quite different in terms of morphology, protein content, and ploidy [43,44]. Some scholars argue that the field has moved beyond the lack of cyst stage as the current animal model induces disease and has provided a significant understanding of parasite virulence, host susceptibility, and disease progression [45]. Currently, mechanisms for the induction of *E*. *histolytica* developmental changes and cyst production are not available. Researchers can obtain cysts from human patients in the clinical setting or nonhuman primates in captivity, which limits their access to most laboratories [46]. Targeting parasite’s developmental changes is a strategy that can result in transmission halting, as only the parasites that are equipped to surviving in the environment, protected by a cyst wall, are infectious to a new host. Approaches focused on encystation are being considered as potential avenues to decline transmission of enteric protozoa (reviewed in [47]). In the 1980s, wet mounts of fecal samples were routinely conducted [24]. Today, there are some diagnostic methods for the identification of the cyst-like structures that are strain specific. The monoclonal antibody 1A4 targets the Jacob2 lectin, while avoiding cross-reactivity with xenic cultures of *E*. *dispar* isolates [48]. Excystation attempts found histamine and glucose availability affect these metabolic processes [49]. The most recent attempt to determine the presence of cysts used a colitis mouse model but found no cysts in tissue histology, cecum, or stool [50]. Having a mouse model that produces infectious cysts that remain stable in storage would be beneficial for the parasitic community for the following reasons: (1) reduces the number of animals used to maintain parasite virulence; (2) alleviates the need for the continuous passage of parasites in culture; (3) improves methods of detection for food safety and patient diagnostics; (4) provides a new platform for antiparasitic drug screening; (5) allows for the study of the host response to developmental changes of the parasite; and (6) permits the examination of *E*. *histolytica’s* interactions with other pathogenic and nonpathogenic protozoans (Fig 1). For drug screening and host immune response studies, a rodent model that can be orally challenged with either trophozoite or cyst stages of the parasite, while displaying invasive colitis, would be the most useful. Particular challenges that would need to be addressed are (1) consistency in the number or cysts used to initiate infection; (2) the extent of invasive disease; and (3) the number of cysts recovered from the colon or fecal samples.
